# Vitamin C and E Treatment Blocks Changes in Kynurenine Metabolism Triggered by Three Weeks of Sprint Interval Training in Recreationally Active Elderly Humans

**DOI:** 10.3390/antiox10091443

**Published:** 2021-09-10

**Authors:** Victoria L. Wyckelsma, Ada Trepci, Lilly Schwieler, Tomas Venckunas, Marius Brazaitis, Sigitas Kamandulis, Henrikas Paulauskas, Helena Gapeyeva, Mati Pääsuke, Stefano Gastaldello, Sophie Imbeault, Håkan Westerblad, Sophie Erhardt, Daniel C. Andersson

**Affiliations:** 1 Department of Physiology and Pharmacology, Karolinska Institutet, 171 77 Stockholm, Sweden; victoria.wyckelsma@ki.se (V.L.W.); ada.trepci@ki.se (A.T.); lilly.schwieler@ki.se (L.S.); stefano.gastaldello@ki.se (S.G.); sophie.imbeault@ki.se (S.I.); hakan.westerblad@ki.se (H.W.); sophie.erhardt@ki.se (S.E.); 2 Institute of Sports Science and Innovations, Lithuanian Sports University, 44221 Kaunas, Lithuania; tomas.venckunas@lsu.lt (T.V.); marius.brazaitis@lsu.lt (M.B.); sigitas.kamandulis@lsu.lt (S.K.); henrikas.paulauskas@lsu.lt (H.P.); 3 Clinic of Medical Rehabilitation, Inpatient Rehabilitation Centre, East Tallinn Central Hospital, 10138 Tallinn, Estonia; helena.gapeyeva@ut.ee; 4 Institute of Sport Sciences and Physiotherapy, Faculty of Medicine, University of Tartu, 50090 Tartu, Estonia; mati.paasuke@ut.ee; 5 Cardiology Unit, Karolinska University Hospital, 171 64 Stockholm, Sweden

**Keywords:** skeletal muscle, mitochondria, antioxidants, reactive oxygen/nitrogen species, endurance training, sprint interval training

## Abstract

The kynurenine pathway (KP) is gaining attention in several clinical fields. Recent studies show that physical exercise offers a therapeutic way to improve ratios of neurotoxic to neuroprotective KP metabolites. Antioxidant supplementation can blunt beneficial responses to physical exercise. We here studied the effects of endurance training in the form of sprint interval training (SIT; three sessions of 4–6 × 30 s cycling sprints per week for three weeks) in elderly (~65 years) men exposed to either placebo (*n* = 9) or the antioxidants vitamin C (1 g/day) and E (235 mg/day) (*n* = 11). Blood samples and muscle biopsies were taken under resting conditions in association with the first (untrained state) and last (trained state) SIT sessions. In the placebo group, the blood plasma level of the neurotoxic quinolinic acid was lower (~30%) and the neuroprotective kynurenic acid to quinolinic acid ratio was higher (~50%) in the trained than in the untrained state. Moreover, muscle biopsies showed a training-induced increase in kynurenine aminotransferase (KAT) III in the placebo group. All these training effects were absent in the vitamin-treated group. In conclusion, KP metabolism was shifted towards neuroprotection after three weeks of SIT in elderly men and this shift was blocked by antioxidant treatment.

## 1. Introduction

Biological ageing involves a progressive decline of cellular function, is a high-risk factor for morbidity, and places individuals at higher risk of diseases [1,2]. Within an increasingly ageing population, actions towards health promotion are critical not only to improve the quality of life for older individuals but also to mitigate escalating health care costs. The kynurenine pathway (KP) is a unique pathway implicated in a high number of pathologies associated with ageing, including those affecting bone, skeletal muscle, cardiovascular, and mental health [3,4,5]. Moreover, changes in KP metabolites both in the cerebrospinal fluid and in serum have been shown to relate to age in healthy individuals as well as in patients with age-related disorders [6,7,8,9,10]. Furthermore, a correlation between changes in KP metabolites and frailty status has been recently shown in older humans [11].

In short, the KP pathway begins with the breakdown of free tryptophan where about 95% of free tryptophan is turned into kynurenine by the enzymes indoleamine 2,3-dioxygenase 1 (IDO1) and tryptophan 2,3-dioxygenase 2 (TDO2). Kynurenine can be metabolized into either the neuroprotective kynurenic acid by kynurenine aminotransferases (KATs) [12] or the free radical generator 3-hydroxykynurenine by kynurenine 3-monooxygenase. 3-hydroxykynurenine is then eventually turned into the neurotoxic quinolinic acid or the protective picolinic acid [13,14]. Importantly, kynurenine and its metabolites—including quinolinic acid and 3-hydroxykynurenine—are strongly correlated to depression, schizophrenia, and other neurodegenerative disorders [15,16,17]. Quinolinic acid in particular is linked with many disorders acting as a neurotoxin, proinflammatory mediator, pro-oxidant molecule, and by affecting the integrity and cohesion of the blood–brain barrier [18].

Chronic low-grade inflammation and a redox imbalance with excessive production of reactive oxygen/nitrogen species (ROS) relative to the antioxidant defense are considered major factors underlying age-related functional impairments [1,19], including impaired skeletal muscle function [1,19], and the generation of quinolinic acid and 3-hydroxykynurenine have been linked to inflammation and oxidative stress [20]. Physical exercise is known to have a positive influence on ageing by reducing chronic low-grade inflammation and negative ROS-induced effects [21]. Intriguingly, the production of ROS is increased during health promoting endurance exercise [22,23] and antioxidant treatment has been shown to reduce beneficial effects of endurance training [24,25,26,27], although other studies report no blunting of exercise-induced adaptations by antioxidant treatment [28,29].

Endurance exercise stimulates mitochondrial biogenesis, including increased expression of mitochondria-targeted KATs that promote the conversion of kynurenine to kynurenic acid [30]. Young adults who engaged in endurance training displayed increased KAT IV and enhanced conversion of kynurenine to kynurenic acid when compared to resistance-type exercise [31,32]. Basal kynurenine metabolites in plasma from older adults have been investigated in a 12-week training study where both resistance exercise (two days per week) and high-intensity exercise (10 × 60 s efforts at ~90% max HR, one day per week) were undertaken [33]. The study did not detect large changes, nonetheless positive trends for decreases in kynurenine and increases in more neuroprotective metabolites were observed [33]. Another study found no improvement in depressed patients levels of kynurenine, kynurenic acid, or their ratios following 12 weeks of low-to-moderate training [34]. However, in that study the KP metabolites were not measured in immediate conjunction to the exercise effort but up to a week after the final training session [34].

Sprint interval training (SIT) is an endurance exercise modality that is short in duration, yet highly metabolically demanding, which has the potential to induce exercise adaptations in a time efficient way. Here, we studied whether KP metabolism was altered in older adults exposed to three weeks of repeated SIT sessions. Given the important role of skeletal muscle kynurenine conversion [30], and that highly trained endurance athletes have more KATs in their muscles than recreationally active people [32], we also assessed if KATs were upregulated following the three weeks of repeated SIT sessions. Finally, due to proposed opposing effects of ROS in ageing (i.e., functional deterioration due to chronic low-grade oxidative stress vs. beneficial effects of transitory ROS increases during physical training sessions), we studied the effects of treatment with the antioxidants vitamin C and E on KP-related responses to SIT. We hypothesize that three weeks of SIT will have positive effects on the balance between neuroprotective and neurotoxic KP metabolites and that it will increase the expression of KATs in muscle, and that these effects will be blunted by the antioxidant treatment.

## 2. Materials and Methods

### 2.1. Volunteers and Training

The volunteers in this study and training protocols have been described before [26]. Briefly, recreationally active male older adults (65.3 ± 1.5 years; *n* = 20) participated in the study; none of the participants were engaged in any structured sport training program but undertook approximately of 4–5 h of exercise a week upon enrolment into the study. The participants were instructed to continue their normal diet and sleeping habits during the study, but these aspects were not strictly controlled or followed up.

Subjects performed three weeks of SIT while being treated with high doses of antioxidants in the form of vitamin C (1 g daily) and vitamin E (235 mg daily; *n* = 11) or placebo (*n* = 9). We used the same doses of vitamins as in previous studies addressing the effects of antioxidants on the response to training [24,25,27]; it should be noted that these doses are about an order of magnitude higher than recommended daily intakes. Treatments were initiated 7 days before the first SIT session to ensure that there was a stable increase vitamin C and E levels prior to exercise interventions. At the time of the study, none of the subjects was diagnosed with a neurological disorder or mental illness. Training adherence was 100% with no adverse events being reported. The study was approved by the Kaunas Regional Biomedical Research Ethics Committee (ethics number BE-2-35) and was in agreement with the Declaration of Helsinki. All participants gave written informed consent before participation.

**Figure 1:**
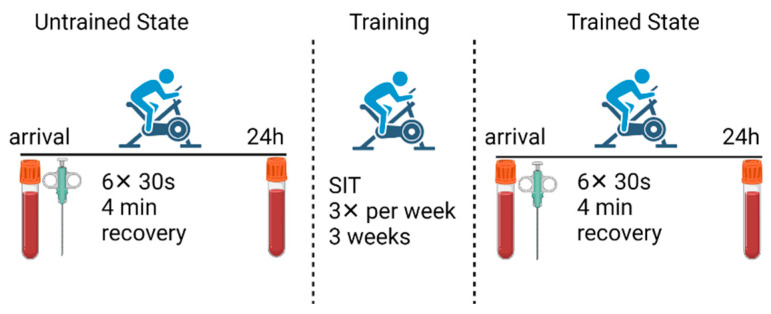
Schematic overview of the study outline. Volunteers undertook the first sprint interval training (SIT) session (untrained state) followed by three weeks of SIT training. The final training session (trained state) identical measures were taken. Tubes and needle indicate taking blood samples and muscle biopsies, respectively.

Training consisted of nine sessions (three sessions a week for three weeks). Each session consisted of a warm-up followed by 4–6 repetitions of 30 s all-out cycling bouts (Wingate tests) with 4 min of rest between bouts [26,35]. A cycle ergometer with continuous power recording was used to quantify the amount of work produced during each SIT session. Vastus lateralis muscle biopsies were taken before the first (untrained state) and before the last (trained state) SIT sessions. Antecubital venous blood samples were collected before and 24 h after the first (untrained state) and the last (trained state) SIT sessions (Figure 1). Note that muscle biopsies and blood samples were taken under resting conditions, i.e., at least 24 h after a SIT session. All testing before and after the SIT were conducted at the same time of day and volunteers were required to maintain their regular diet including replicating diet in the 24 h prior to each testing session.

### 2.2. Plasma Preparation

Plasma samples were collected in vacuum tubes using EDTA as an anticoagulant (EDTAK3, 3 mL), mixed gently by inverting 8–10 times, and kept at 2–8 °C until centrifugation. Blood samples were centrifuged at 1200× *g* for 15 min within 30 min after blood collection. Plasma was separated from the red blood cells as soon as possible (maximum, 10–15 min) after centrifugation and kept at −80 °C until analysis. 30 μL of plasma sample was mixed with 30 μL of internal standard 0.5 μM in 10% ammonium hydroxide (UPLC grade) solution and vortexed for 15 s. 60 μL of 200 nM ZnSO_4_ (+5 °C) was added and vortexed for 15 s. 30 μL of methanol (+5 °C) (UPLC grade) was added and vortexed for 15 s. The mixture was centrifuged for 10 min at 2841× *g* at room temperature. 30 μL of the supernatant was mixed with 30 μL of formic acid 5% in LC-MS Certified Clear Glass 12 × 32 mm vials (Waters, Manchester, UK, product no. 186005662CV). Vials were transferred to an autosampler (set to 5 °C) that injected 1.5 μL into the ultra-performance liquid chromatography tandem mass spectrometry (UPLC–MS/MS) system [36].

### 2.3. Ultra-Performance Liquid Chromatography/Mass Spectrometry (UPLC-MS/MS)

Tryptophan, kynurenine, kynurenic acid, quinolinic acid, 3-hydroxykynurenine, and picolinic acid in plasma were analyzed simultaneously using UPLC-MS/MS system using a Xevo TQ-XS triple-quadrupole mass spectrometer (Waters, Manchester, UK) equipped with a Z-spray electrospray interface and a Waters Acquity UPLC I-Class FTN system (Waters, MA, USA). The MS was operated in electrospray-positive multiple reaction monitoring (MRM) mode. The conditions were set as follows: interface: electrospray; source temperature of 150 °C; desolvation gas flow rate 1000 L/h; cone gas flow rate: 150 L/h; capillary voltage of 3.0 kV; desolvation temperature 650 °C; and detector gain 1. The column used was Acquity HSS T3 1.8 μm with dimensions 2.1 × 150 mm, (Waters, Manchester, UK, serial number: 186003540) and set to a temperature of 50 °C. In addition, a guard column (Waters, Manchester, UK, Vanguard HSS T3 1.8 μm 2.1 × 50 mm column, PN: 186003976) was installed to retain contaminants from the mobile phase. The two used mobile phases consisted in: solvent A: 0.6% formic acid in water (UPLC grade) and solvent B: 0.6% formic acid in methanol (UPLC grade). The flow rate used was 0.3 mL/min and the run time for each sample was 13.0 min. For more detailed and method validation see [36].

KP metabolites show large variations between individuals at rest and over time in connection with exercise [36,37]; individual data of KP metabolites in association with the first SIT session in the placebo group have been published [36]. Here we focused on KP metabolite levels under resting conditions and to get robust values, we use the mean of measurements preformed in each individual before and 24 h after SIT sessions in the untrained and trained state, respectively. In a few cases, successful measurements were not obtained both before and 24 h after exercise and only one value was then used.

### 2.4. Muscle Biopsies

We used previously described and validated procedures for taking needle muscle biopsies [38]. Briefly, after skin sterilization and local anesthesia, a 1–2 mm-long skin cut was made with the tip of a scalpel. Biopsies were collected from vastus lateralis muscles using an automatic biopsy device (Pajunk DeltaCut, Geisingen, Germany). A 14-gauge disposable needle was inserted through the cut, perpendicular to the muscle fibers, until the fascia was pierced. Two to three samples (~15 mg each) were collected from one puncture site at each time point. A local compression was then applied on the biopsy site for a few min. Muscle samples were immediately frozen in liquid nitrogen and stored at −80 °C.

### 2.5. Western Blotting

Approximately 10 mg of frozen muscle was weighed and homogenized on ice (1:20 *w*/*v*) in HEPES lysis buffer (20 mM HEPES, 150 mM NaC1, 5 mM EDTA, 25 mM KF, 5% Glycerol, 1 mM Na_3_VO_4_, 0.5% Triton, pH 7.6) with Protease Inhibitor (no. 11836145001, Roche, 1 tablet per 50 mL). Homogenate was diluted to 33 μg wet weight muscle per μL using 3 × SDS denaturing solution (0.125 M Tris-HCI, 10% glycerol, 4% SDS, 4 M urea, 10% 2-mercaptoethanol and 0.001% Bromophenol Blue, pH 6.8). Finally, samples were further diluted to 2.5 μg wet weight muscle per μL with 1 × SDS solution (3× SDS denaturing solution diluted 2:1 with 1 × Tris.Cl (pH 6.8)). A calibration curve with muscle samples from healthy young adults (18–35 years) was run on every gel for western blotting. All samples were stored at −80 °C until analysis.

10 µg of protein was separated on either 18 or 26 well 4–15% TGX stain-free gels, after separation gels had total protein visualized prior to transfer and analysis on Image Lab software (Image Lab 6.0, Bio-Rad, Hercules, CA, USA). Protein was wet transferred to polyvinylidene fluoride (PVDF) membrane followed by blocking at room temperature for 2 h using LI-COR Intercept buffer in phosphate-buffered saline (PBS). After blocking, membranes were incubated in primary antibody overnight at 4 °C and for 2 h at room temperature. Primary antibody details are as follows: KAT I (anti-CCBL1, 121156-1-AP, Proteintech, 1:1000), KAT III (anti-CCBL2, HPA026538, Atlas Antibodies, Stockholm, Sweden. 1:200), KAT IV (GOT2. ARP43518_T100, Aviva systems biology, 1:1000), and TDO2 (15880-1-AP, Proteintech, 1:1000). All antibodies were diluted in LI-COR Intercept buffer in PBS, 1:1 *v*/*v* with 1 × tris-buffered saline with Tween (TBST). After incubation in primary antibody, membranes were washed in 1 × TBST, incubated in secondary antibody (1:20,000, IRDye 680-conjugated donkey anti-mouse IgG and IRDye 800-conjugated donkey anti-rabbit IgG (926–68,072; 926–32,213; LI-COR Biosciences) and immunoreactive bands were visualized using infrared fluorescence on an IR-Odyssey scanner. Band densities were analyzed using Image Studio Lite v 5.2 (LI-COR Biosciences, Lincoln, NE, USA). During data analysis, the density of each given protein was measured relative to the calibration curve and then normalized to the total protein as measured for each lane in stain-free gels. The same calibration curve was used across all gels and data are expressed relative to the average of the pre training biopsies from each group on each gel.

### 2.6. Statistical Analysis

Data are presented as mean ± SEM. Statistical analyses were performed with GraphPad Prism 8 (Graphpad Software Inc., San Diego, CA, USA) Statistically significant differences were analyzed by a two-way repeated measures analysis of variance (ANOVA), which was followed by Tukey’s post-hoc test when an effect of training and/or treatment (placebo vs. vitamin) was observed. Significance was set at *p* < 0.05.

## 3. Results

### 3.1. KP Metabolites in Plasma at Rest in the Untrained and Trained States

**Figure 2:**
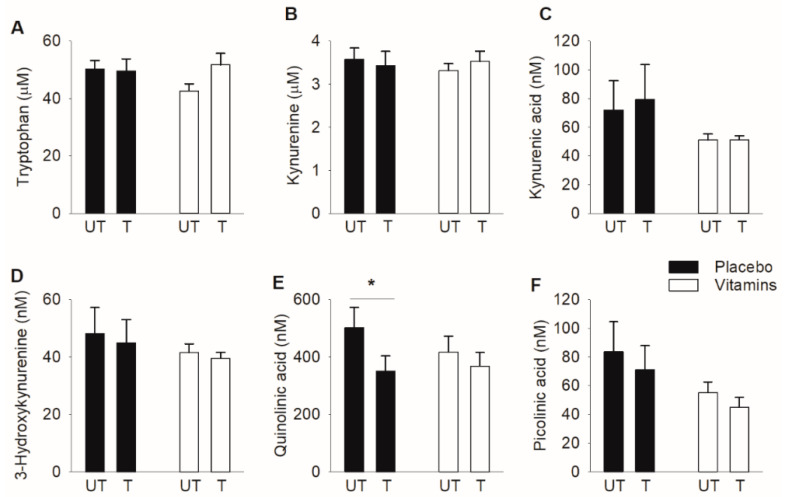
Effects of three weeks of SIT with or without antioxidant supplementation on KP metabolites in plasma of resting individuals. (**A**) Tryptophan, (**B**) kynurenine, (**C**) kynurenic acid, (**D**) 3-hydroxykynurenine, (**E**) quinolinic acid, and (**F**) picolinic acid in plasma in connection with the first (untrained, UT) and the last (trained, T) SIT sessions. Black bars, placebo group (*n* = 9) and white bars, vitamin-treated group (*n* = 11). Data presented as mean ± SEM. * *p* < 0.05 between untrained and trained state with two-way repeated measures ANOVA followed by Tukey’s post-hoc test.

To assess whether three weeks of SIT alters plasma KP metabolites in the rested state and if this was affected by vitamin C and E supplementation, we measured six KP metabolites in connection with the first (untrained state) and the final (trained state) SIT sessions. The results show a statistically significant reduction of the neurotoxic quinolinic acid in the trained state in the placebo group, whereas this KP metabolite was not affected by training in the vitamin-treated group (Figure 2).

**Figure 3:**
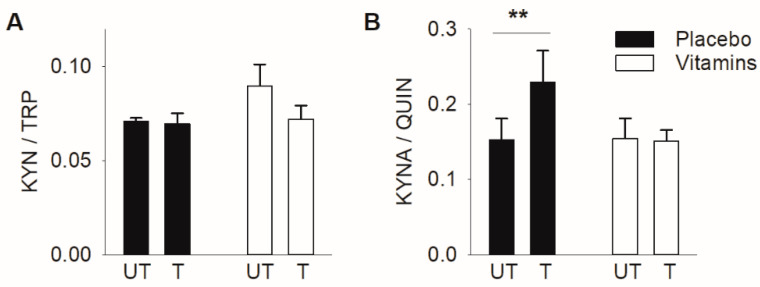
Three weeks of SIT increased the kynurenine acid to quinolinic acid ratio only in the placebo group. (**A**) The kynurenine to tryptophan (KYN/TRP) and (**B**) kynurenine acid to quinolinic acid (KYNA/QUIN) ratios in plasma in connection to the first (untrained) and last (trained) SIT sessions. Black bars, placebo group (*n* = 9); white bars, vitamin-treated group (*n* = 11). Data presented as mean ± SEM. ** *p* < 0.01 between untrained and trained state with two-way repeated measures ANOVA followed by Tukey’s post-hoc test.

The plasma kynurenine to tryptophan (KYN/TRP) ratio has been used to assess the activity in the initial step of the KP [39]. We observed no differences in the KYN/TRP ratio either between the untrained and trained states or between placebo and vitamin-treated subjects (Figure 3A). Another commonly used measure is the ratio between the neuroprotective kynurenic acid and the neurotoxic quinolinic acid (KYNA/QUIN) [32,40,41,42]. In the placebo group, the KYNA/QUIN ratio was ~50% higher in the trained than in the untrained state, whereas it was not affected in the vitamin-treated group (Figure 3B). Thus, three weeks of SIT appeared to induce KP-related neuroprotection in the placebo group but not in the vitamin-treated group.

### 3.2. KAT Protein Expression in Muscle following Three Weeks of SIT

**Figure 4:**
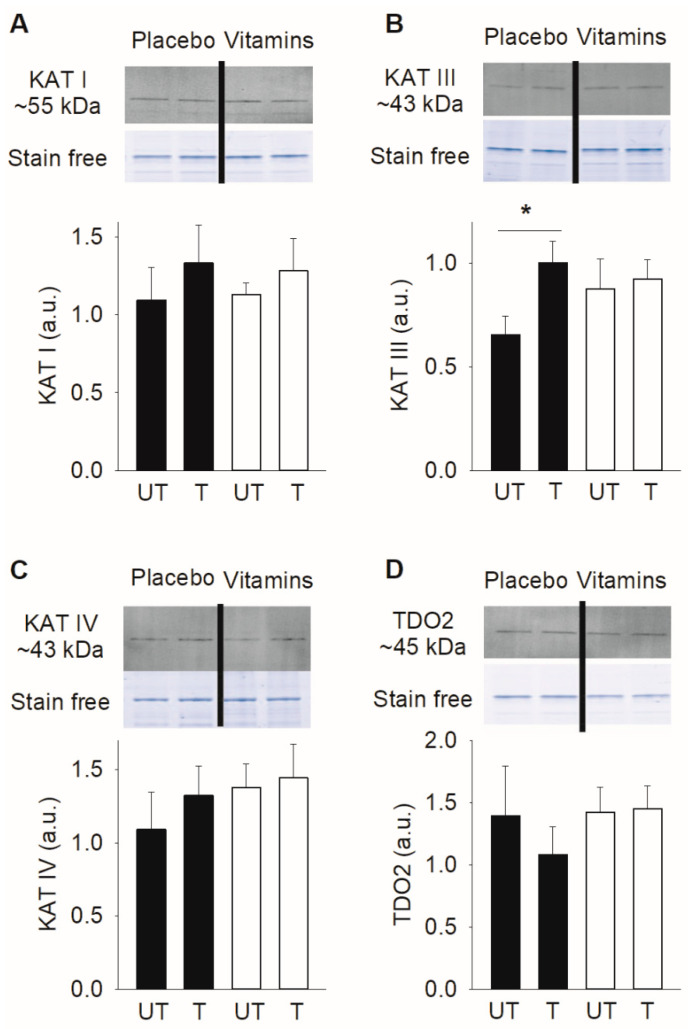
Three weeks of SIT increased KAT III protein expression in the placebo but not in the vitamin-treated group. Mean data (± SEM) and representative blots for (**A**) KAT I, (**B**) KAT III, (**C**) KAT IV, and (**D**) TDO2 in skeletal muscle biopsies taken under resting conditions in the untrained (UT) and trained (T) states. Black bars, placebo group (*n* = 7–8); white bars, vitamin-treated group (*n* = 8–9). Stain free images were used as total protein loading controls and the actin band in such stain free images reflected the total protein content and are shown below the Western blots. Each protein was normalized against its own calibration curve (~5–40 µg wet weight protein) and expressed relative total protein loading. * *p* < 0.05 between untrained and trained state with two-way repeated measures ANOVA followed by Tukey’s post-hoc test.

We previously showed that KAT I-IV expression is higher in muscle of trained endurance cyclists compared to recreationally active individuals [32], hence indicating a positive effect of endurance training on KAT expression. However, in the present study, investigating older subjects, we only observed a higher expression KAT III in the trained than in the untrained state in the vitamin-treated group, whereas all other KAT measurements showed no difference between the two states (Figure 4). Moreover, the protein expression of muscle TDO2, which facilitates the conversion of tryptophan to KYN, was not changed following training in either group.

## 4. Discussion

We here tested the hypothesis that three weeks of endurance training in the form of repeated SIT sessions would have a positive effect on KP metabolism in elderly men and that this effect would be blunted by antioxidant treatment with vitamin C and E. In accordance with our hypothesis, the results show decreased blood plasma concentration of the neurotoxic quinolinic acid and an increased muscle protein expression of KAT III in the placebo group but not in the vitamin-treated group.

Chronic inflammatory mechanisms elevate several KP intermediates, including quinolinic acid, as shown by elevated quinolinic acid levels found in cerebrospinal fluid and post-mortem brain tissue of patients with neuroinflammatory disorders [43]. Furthermore, increased quinolinic acid is linked to anxiety in animal models [44] and a reduction in the kynurenic acid/quinolinic acid ratio is commonly observed in depressed patients [40,41,42]. Intense physical exercise can induce acute muscle inflammation. However, a well-described benefit of regular physical exercise is its systemic anti-inflammatory effects, which are not exclusive to skeletal muscle [45]. In the present study, we measured plasma KP metabolites under resting conditions before (untrained state) and at the end (trained state) of the three weeks of repeated SIT sessions, which promote ROS-dependent inflammatory signaling [26]. Our results show that in the placebo group, the quinolinic acid plasma concentration was decreased by ~30% and the kynurenic acid/quinolinic acid ratio was ~50% higher in the trained state. Thus, these results imply that the three weeks of SIT triggered mental health-promoting changes KP metabolism. In this context, it is intriguing that the effect was obtained with a total of only 7–9 min of high-intensity exercise per week. Furthermore, the effect seems robust in that it was statistically significant with the limited number of individuals included in the study and without full control of diet and sleep, factors that can affect the concentration of KP metabolites [46,47].

In contrast to the placebo group, we observed no potentially beneficial training effect on KP metabolism in the vitamin-treated group, which fits with previous studies showing that antioxidant supplementation can hinder benefits of physical exercise [24,25,26,27,48]. Thus, the results of our study adds effects on KP metabolism to the general concept of previous studies describing intricate effects of ROS, where transient and over-time-limited ROS challenges can be beneficial whereas prolonged ROS increases are likely to be detrimental [49,50].

The conversion of kynurenine to kynurenic acid is catalyzed by four KATs. Here, we measured the protein expression of three of these (KAT I, III, and IV), of which KAT III and IV are targeted to the mitochondria [4,12,30]. We previously showed that, despite a generally higher mRNA expression of genes encoding mitochondria-related proteins in the placebo than in the vitamin-treated group after three weeks of SIT, the expression of mitochondrial electron complex proteins only displayed a non-significant tendency towards being higher in the placebo group and the maximal oxygen uptake during cycling remained unchanged [26]. Nevertheless, we here show an approximate 50% increase in KAT III protein expression in trained placebo-exposed subjects, whereas the three weeks of SIT had no effect on KAT III protein expression in the vitamin-treated group. This result broadly fits with the observed changes in KP metabolites (decreased quinolinic acid levels and increased kynurenic acid to quinolinic acid ratio), where an increased KAT activity would shift the KP balance to kynurenine being converted to kynurenic acid rather than being further metabolized to quinolinic acid. The lack of effect of SIT on the protein expression of KAT III in the vitamin-treated group provides yet another example of a training response that is hampered by antioxidant treatment [25,26,27].

We observed no effect of the three weeks of SIT on the protein expression of TDO2, which is one of the enzymes that catalyzes the conversion of tryptophan to kynurenine, in either the placebo or vitamin-treated groups. In accordance with this result, we did not observe any training-induced effects on the plasma levels of tryptophan or kynurenine or the ratio between these two KP metabolites.

High-intensity interval training has proven to be an effective and time-efficient training regimen for healthy individuals, as well as for individuals suffering from diseases that limit exercise performance [51]. Recent meta-analyses show improved aerobic exercise performance in young sedentary or recreationally active individuals after a period of high-intensity interval training similar to that used in the present study [52,53,54]. However, the SIT regimen used in the present study did not improve the aerobic capacity of the elderly participants [26] and the present effects on KP metabolism were relatively small. It has previously been show that nine SIT sessions effectively increases the aerobic capacity of elderly individuals [55]. In that study, the nine SIT sessions were performed over six weeks compared to three weeks in the present study. Thus, larger effects on KP metabolism than those reported here might be expected with a longer total training period than three weeks and/or with longer rest periods between training sessions [26].

## 5. Conclusions

**Figure 5:**
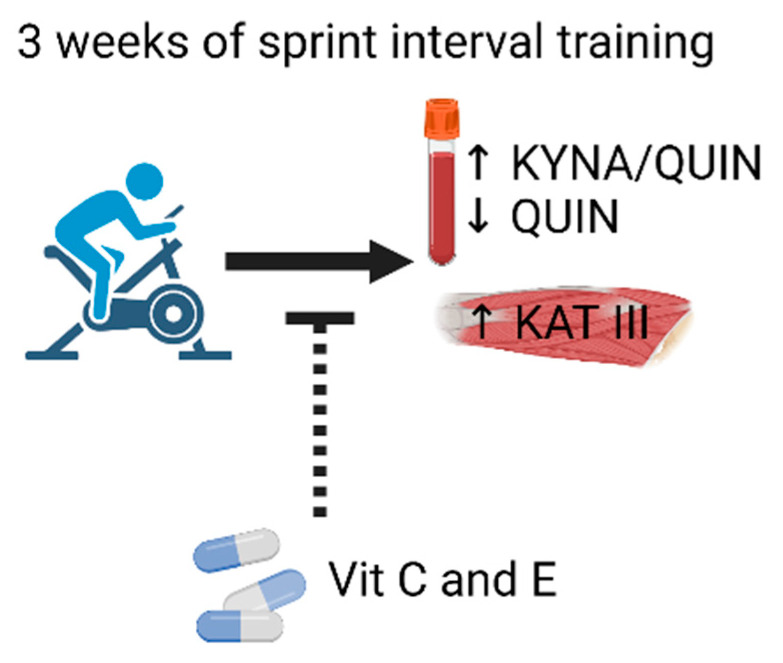
Graphical summary of major results. Three weeks of SIT altered important aspects of kynurenine metabolism: decreased quinolinic acid levels and increased kynurenic acid to quinolinic acid ratio in plasma and increased KAT III expression in skeletal muscle. Per oral administration of the antioxidant vitamins C and E, prior to and during the exercise period, blocked these potentially neuroprotective training effects.

The KP metabolism was shifted in a neuroprotective direction after three weeks of SIT, with a total of less than 30 min of high-intensity exercise, in recreationally active elderly men and this shift was blocked by antioxidant treatment with vitamin C and E (Figure 5). Thus, transient ROS increases during SIT sessions emerge as important triggers of beneficial adaptations and this signaling is disturbed by antioxidant treatment. Still, the effect was relatively modest and future studies where individuals perform SIT over a longer time frame and/or with longer rest periods between SIT sessions are required to establish whether this type of training effectively promotes beneficial changes in KP metabolism in an elderly population.

## Data Availability

Data is contained within the article.
